# The Urinary Microbiome; Axis Crosstalk and Short-Chain Fatty Acid

**DOI:** 10.3390/diagnostics12123119

**Published:** 2022-12-10

**Authors:** Hee Jo Yang, Doo Sang Kim, Kwang Woo Lee, Young Ho Kim

**Affiliations:** Department of Urology, Soonchunhyang University School of Medicine, Cheonan 31151, Republic of Korea

**Keywords:** microbiota, brain-gut axis, inflammasomes, fatty acids

## Abstract

Our knowledge that “urine is sterile” is no longer accepted after the development of a next-generation sequencing (NGS) test. Using NGS, microbiota in the human body were discovered, and it is expected that this will improve our understanding of human diseases. However, the mechanism involved in the effect of the microbiome on diseases is still poorly understood. Associations of gut microbiome with diseases have been recently reported. Based on such associations, bladder–gut–brain axis, gut–bladder axis, gut–vagina–bladder axis, and gut–kidney axis as novel mechanisms of action of the microbiome have been suggested. Each axis can influence the development and progression of disease through interactions. In these interactions, metabolites of the microbiome including short-chain fatty acids (SCFA) and the inflammasome play an important role. Inflammasomes are multiprotein oligomers that can initiate inflammatory responses. Inflammasomes can trigger inflammation and pyroptosis and ultimately contribute to disease development. SCFAs play an important role in immune cell migration, cytokine production, and maintenance of cellular homeostasis. Associations of inflammasomes with systemic diseases such as obesity and insulin resistance have been reported. The roles of inflammasomes and SCFAs in kidney, bladder, and prostate diseases have also been revealed recently.

## 1. Introduction

After next-generation sequencing (NGS) tests revealed that the urine was not sterile [1], several microbes were identified. It is assumed that these microbes may be pathogens, and many studies have been conducted using NGS for urological diseases with unknown causes [2].

If urine is not sterile like feces with many pathogens present, it could affect the bladder, and a disease with similar mechanisms to intestinal disease might occur in the urinary system [3]. This suggests that persistent pathogen influx from the intestine to urine, especially in the bladder, where urine is continuously collected, might result in a disease that is refractory to treatment. This hypothesis is the axis theory that is attracting attention these days [4]. Perez-Carrasco et al. have found that 562 species of microbes are present in urine, showing differences in microbial species between males and females. In particular, in the case of women, it has been reported that 62.5% of intestinal-derived species and 32% of vaginal-derived species are shared with species in urine [5]. This makes it possible to speculate that the previously unclear origin of the urine microbiome is the gut. Indeed, the decreased incidence of urinary tract infection (UTI) after transplantation of fecal xenobiotics supports this hypothesis [6]. A mutual symbiotic relationship of these strains suggests that diseases can occur due to crosstalk between each axis.

In UTI, contamination of the periurethral space (perineum) by specific uropathogens present in the gut can occur [7], providing good evidence of an association between the gut and bladder. Thus, it is important to study the relationship between gut microbiota and the development of bacteriuria and UTIs. Recent diagnostic and treatment paradigms for UTIs have focused on symbiosis of the microbiome in a healthy urogenital tract. A recent study has suggested that vaginal bacterial imbalance could also cause UTI [8].

Leue et al. [9] have reported that functional bladder diseases (e.g., overactive bladder (OAB), interstitial cystitis/bladder pain syndrome (IC/BPS), and chronic prostatitis/chronic pelvic pain syndrome (CP/CPPS)) and bowel disease are interconnected. To explain this, the bladder–gut–brain axis (BGBA) hypothesis was proposed [9]. It suggests that experiencing emotional and physical distress in response to threats such as childhood difficulties or traumatic events such as infectious diseases may falsify these risk alerts and that the resulting neuroticism might be a risk factor in the future. In addition, threats such as infection can affect body–brain crosstalk and disrupt patient’s alarm of danger. This affects mood, cognition, and behavior, leading to an imbalance of homeostasis which can cause various diseases [9]. From a microbiome research point of view, dysbiosis of gut might cause mutual diseases in the brain, bladder, and gut.

Recent studies on more axes in connection with various diseases have been presented [10,11,12,13]. In these interactions, inflammasomes, multiprotein oligomers that can initiate inflammatory responses and metabolites of the microbiome such as short-chain fatty acids (SCFA), play an important role. Inflammasomes are pattern recognition receptors, which elicit the innate immune response to target pathogens and trigger inflammation [14]. Inflammasome dysfunction may be responsible for underlying inflammatory diseases, which include renal and urological pathologies because many urological diseases and disorders affecting the bladder, prostate, or kidneys are mediated by inflammation. SCFAs, the byproducts of dietary fiber in intestine during fermentation, are the most important energy substances of the epithelial lining of the colon [15]. SCFAs are known for their beneficial effects on intestinal barrier function, systemic anti-inflammatory effects, and their role in producing a fraction of the daily energy requirement of gut microbiota, and recently their role in urological disease has also been highlighted [16,17]. We briefly summarize studies reported so far on various axes related to the occurrence of urinary tract disease and the inflammasome and SCFAs supporting them.

## 2. Axis Crosstalk

### 2.1. Bladder–Gut–Brain Axis

**Figure 1:**
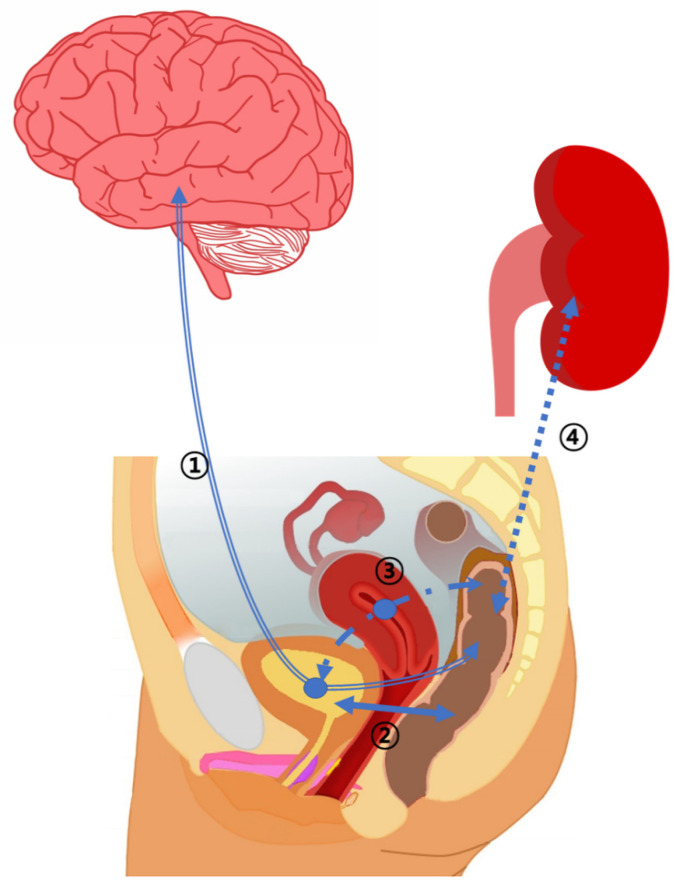
Bidirectionally communication between the gut, bladder, kidney, vagina, and brain. (1) Gut–bladder–brain axis, (2) Gut–bladder axis, (3) Gut–vagina–bladder axis, (4) Gut–kidney axis.

Bladder disorders are linked to various axes. However, the number of studies related to the microbiome is still limited. Leue et al. have proposed the bladder–gut–brain axis (BGBA) hypothesis, suggesting that mental problems and external/internal problems such as infections might cause bladder dysfunction [9] (Figure 1). In particular, dysfunction of the urinary tract often occurs together with dysfunction of the gastrointestinal tract, which can cause serial abnormalities throughout the system. This coexisting urinary and gastrointestinal dysfunction can be a danger signal to the body’s defense system. BGBA is a useful framework to study these interactions [9]. This hypothesis is very important in that microbiome exists in urine [1]. This is because mental problems and infection can also lead to bladder dysbiosis (evidence). Causes of functional urological disease, such as overactive bladder (OAB), interstitial cystitis/bladder pain syndrome (IC/BPS), and chronic prostatitis/chronic pelvic pain syndrome (CP/CPPS) remain unknown. Research on crosstalk of the bladder–gut–brain axis (BGBA) might help us understand these diseases in the future.

### 2.2. Gut–Bladder Axis

There is increasing evidence that intestinal dysbiosis affects end organs. The axis theory has been studied a lot in an effort to understand this interaction [18,19]. Although the gut–bladder axis has not yet been well-characterized, it has been recently reported that recurrent UTI and intestinal dysbiosis are related [11,20] (Figure 1). Another study has reported that abundance of enteric uropathogens is a risk factor for urinary tract infection in kidney transplant patients [10]. Tariq et al. have reported that fecal material transplantation for treating *Clostridum difficile* infection is effective in reducing the frequency of recurrent UTI. The antibiotic susceptibility profile of the organism causing UTI is also improved [6]. Recently, Hultgren et al. have conducted a gut–bladder axis study and suggested that women with recurrent UTI can fall into a vicious cycle where antibiotics administered to eradicate one infection are more likely to cause another infection because the pathogen that does not die can causes re-infection [21]. They proposed three hypotheses regarding the role of the gut: (a) gut microbiota do not directly affect UTI risk, serving only as a passive reservoir for uropathogenic *Escherichia coli* (UPEC) (gut as bystander); and (b) gut microbiota provide a differentially hospitable environment for UPEC, thus modulating the risk of gut colonization and subsequent successful colonization of the bladder (gut as facilitator); and/or (c) host-microbiota interactions in the gut can affect the systemic immune system and cause differential response to bacterial invasion of the bladder (gut as agitator) [21].

### 2.3. Gut-Vagina-Bladder Axis

The etiology of urinary tract infection has been considered as an infection caused by certain urinary tract pathogens present in the gut. This has recently been challenged by the discovery of microbiome in the genitourinary tract. It also suggests that changes in the microbiome might also occur due to dysbiosis in the vagina [8]. Another hypothesis, the gut–vaginal–bladder axis, is that quorum sensing and poly-infection exist between the strain that enters the bladder from the gut and causes UTI and the strain that enters the vagina and enters the bladder causing UTI, with *Gardnerella vaginalis* playing an important role (Figure 1). Kim and Yoo et al. have recently found that three different sub-urotypes in the bladder through a study of 78 patients with recurrent cystitis [22]. The three sub-urotypes are: (1) recurrent UTI (rUTI) could occur in an environment in which UPEC is dominant; (2) rUTI could occur in an environment in which *Gardnerella vaginalis* is dominant; and (3) cystitis could occur in an environment in which *Lactobacillus* is dominant [22].

It has also been reported that *Gardnerella vaginalis* is a very important causative factor for urge incontinence [23]. It has been confirmed that *Gardnerella vaginalis* can strongly induce Ca^2+^ influx and contraction in bladder smooth muscle. In other words, it has been suggested that the microbiome normally present in the vagina may also affect urge incontinence and overactive bladder [23].

### 2.4. Gut–Kidney Axis

The gut microbiota can dynamically affect metabolism and the functioning of the immune system and establish strong connections between bacteria and host systems. An imbalance in the microbiome can result in disruption of the intestinal barrier, which in turn causes an imbalance in the microbiota. It has been recently reported that disruption of the gut microbiota may also result in bacterial passage into the kidney, like the gut–bladder–brain axis, gut–bladder axis, and gut–vagina–bladder discussed earlier [24] (Figure 1). Several well-known uremic toxins, such as ammonia, are produced by microorganisms. In CKD patients, uremic toxin-producing microorganisms are increased whereas SCFA-producing microorganisms are decreased [12]. Microbiota metabolites such as indole-3-acetic acid and p-Cresol sulfate can induce inflammation. Their association with pathological progression of CKD patients has also been confirmed [25,26]. In a study comparing fecal SCFA according to CKD grade, the concentration of butyrate is decreased significantly as renal function deteriorates. Although change in concentrations of acetate and propionate acetate did not show a significant difference, they showed a decreasing trend [13]. In addition, decreases of SCFA-producing strains, *Butyricicoccus* spp., *Faecalibacterium prausnitzii,* and *Roseburia* spp., were identified. These decreases were associated with a decrease of each SCFA in CKD patients [13]. The pathogenic interconnection between gut microbiota and kidney disease is referred to as the gut–kidney axis.

Therapeutic effect of using SCFA in a rat model of renal ischemic-reperfusion injury (IR injury) has been reported. After IR injury, injection of acetate, propionate, and butyrate reduced kidney damage [27], similar to treatment with acetate-producing bacteria, *Bifidobacterium adolesentis* and *B. longum*. The treatment reduced proinflammatory cytokines and chemokines. Sun Y et al. [28] have investigated changes in renal function by injecting butyrate after IR injury. After IR injury, SCFA was increased whereas blood creatinine levels were decreased, suggesting a negative correlation between SCFA and decreased renal function after IR injury. This effect persisted for 7 days after IR injury. Histopathological examination also confirmed that the pathological damage caused by IR injury was decreased when butyrate was injected. Similar results were also observed in IR injury models in other tissues such as intestine, suggesting that the underlying protective mechanisms might be common across tissues [29].

## 3. Substances Involved in Crosstalk

### 3.1. Inflammasome and Infection: Urologic Disease

Inflammation is regulated by the host as a protective response that is activated in response to noxious stimuli such as irritants and pathogens [30]. Inflammasomes are multiprotein oligomers responsible for initiating the inflammatory response. When inflammasome is activated, it induces proteolytic cleavage of pro-capsase1 to generate caspase-1. Inflammasomes can promote maturation and secretion of proinflammatory cytokines interleukin 1β (IL-1β) and interleukin 18 (IL-18) and promote pyroptosis programmed into proinflammatory cell death distinct from apoptosis [31,32]. Pyroptosis in turn releases ATP, uric acid, and intracellular damage-associated molecular patterns (DAMPs) that further propagates inflammation [32].

Recent studies have shown that the inflammasome is associated with the initiation and progression of several diseases, including metabolic diseases such as diabetes and type 2 diabetes, cardiovascular diseases, inflammatory diseases such as multiple sclerosis, and neurological diseases such as Parkinson’s disease [30]. Among them, the NLRP3 inflammasome is the best characterized, which is a family of nucleotide-binding and oligomerization domain-like receptors (NLRs). The NLRP3 inflammasome is located within the cell. It can recognize two types of signals: pathogen-associated molecular patterns (PAMPs) and DAMPs [31,33]. Inflammasomes are expressed in immune and non-immune cells, which contribute to the detection of pathogens and danger signals. They can induce inflammation and pyroptosis, which are involved in the pathogenesis of several diseases.

NLRP3 activation contributes to immune/inflammatory responses in brain disorders. NLRP3 is a bacteria-immune sentinel, involved in the regulation of the gut–brain axis. High levels of intestinal SCFAs activate NLRP3 inflammasome in the intestinal epithelium by binding to G-protein-coupled receptor 43(GPR43) and GPR109A [34]. This activation of inflammasome contributes to intestinal homeostasis and releases IL-18, which plays a protective role in the intestine. Intestinal metabolites can be freely passed through the blood–brain barrier (BBB), thereby regulating brain physiology [35]. This BBB integrity could be modulated by characteristic components of the microbiome that mediate more microbial signaling from the gut to the brain. In germ-free mice, BBB may be weakened due to the lack of intestinal microbiota [36]; however, colonization of butyrate-producing bacteria can restore a weakened BBB [35]. There is consistent evidence that bidirectional interactions between inflammasome multiprotein complexes and the gut microbiota contribute to the maintenance of gut homeostasis [37]. Bidirectional interactions between gut microbiota and the NLRP3 inflammasome appear to contribute to preserving homeostasis of the gut microenvironment [38].

These inflammasomes can mediate various urological pathologies. Inflammasome activity and chronic inflammation may mediate benign and malignant pathologies (e.g., IC/BPS, UTI, benign prostatic hyperplasia or prostate cancer) [39]. Kummer et al. [40] confirmed the presence of NLRP3 in the human bladder in an early study, and Hughes et al. [41] confirmed that NLRP3 in mice is localized in the urothelial layer. The NLRP3 inflammasome was first discovered to mediate bladder inflammation in a model of cyclophosphamide-induced hemorrhagic cystitis [42]. In the case of IC/BPS, due to bladder lining defects, it is presumed that urinary solutes or some molecules (e.g., derived from damaged cells) that have penetrated the bladder wall will activate the inflammasome. This multiprotein complex can produce bioactive interleukins, which in turn can enhance the inflammatory response. Eventually, more cells are damaged and a process occurs to release the next DAMP [43]. Inflammasomes also act on the UTI. UPEC produces lipopolysaccharide (LPS), flagellin, and a-hemolysin, which are PAMPs that activate the NLR inflammasome. Injection of LPS into the bladder wall induced irritable voiding, a symptom of UTI, which was blocked by glyburide. This makes it possible to presume that NLRP3 is involved in the onset of discomfort symptoms caused by UTI [42,44]. Likewise, inflammasomes might play a role in inflammation caused by prostate microbiota. According to a recent study, inflammasome-mediated inflammatory processes can lead to storage/voiding symptoms, bladder fibrosis, and denervation [32]. Inflammasome activation induced by bladder outlet obstruction (BOO) involves DAMP. It is induced by hypoxia/reperfusion, increased intravesical pressure and repetitive stretching of the bladder wall. BOO triggers an inflammatory process mediated by NLRP3, leading to negative consequences such as fibrosis. In a study by Hughes et al. [44], treated with NLRP3 inhibitor glyburide in mice of the BOO model, they found that NLRP3 was activated during the acute phase of BOO and, consequently, caused inflammation and bladder enlargement. Additionally, the inhibition of NLRP3 with glyburide or IL-1β with a receptor antagonist (anakinra) blocked the appearance of fibrotic endpoints [45]. Eventually, benign prostate hyperplasia (BPH) could be a result of inflammasome activation [31,46].

Numerous as yet unknown mechanisms exist for the hidden influence of the gut microbiota in the development of disease. One study on the inflammasome pathway in the brain has found that gut microbiota can modulate pathways affecting neuroinflammatory conditions such as Parkinson’s disease, brain function, and depressive and anxiety-like behaviors through inflammasome signaling [47]. In addition, it has been confirmed that the NLRP3 inflammasome can strongly act as a major amphipathic mediator of the intestinal–lung axis [48]. Understanding the role of the inflammasome in various pathological conditions including urologic disease and neurological disease, we will be able to target it and find treatments to prevent these diseases and their symptoms.

### 3.2. SCFAs (Short Chain Fatty Acids)

The gut is a digestive system of humans. It is also an important component of the immune system. In particular, the importance of gut microbiota has been recently emphasized because its metabolites, hormones, cytokines, and SCFAs can regulate local and systemic immunity [49] (Table 1). Crosstalk between the gut microbiome and immunity is important for maintaining immune homeostasis. [50].

SCFAs are a kind of metabolites of microbiota. They are fatty acids with fewer than six carbon atoms. SCFAs are formed by the breakdown of indigestible dietary substrates by the human gut microbiota, along with other volatile fatty acids and alcohols. Acetate, propionate, and butyrate are major SCFAs found mainly in the proximal colon at concentrations of 50–120 mM [55]. SCFAs are of great interest in microbiome research because of their beneficial effects on intestinal barrier function, systemic anti-inflammatory effects, and their role in producing a fraction of daily energy requirement of gut microbiota. SCFAs are produced by two major groups of bacteria. Propionate and acetate are produced by *Bacteroidetes* and butyrate is produced by *Firmicutes* [56].

Changes in the host’s dietary pattern can cause significant changes in the composition of gut microbiota, leading to changes in the production of microbial metabolites [57]. In fact, it has been confirmed that suppressing production of SCFAs production using a low-fiber diet or increasing microbial production of SCFAs using a high-fiber diet can result in rapid changes in SCFAs in the cecum and serum. Although acetate is generally the most abundant SCFAs in the cecum, the ratio of acetate:butyrate:propionate reported in several studies varies widely [58,59]. Additionally, these ratios are changed dramatically by diet manipulation [57].

SCFAs also play an important role in the immune system. SCFA has regulatory capacity for various immune cells such as regulatory T cells (Tregs), macrophages, antigen-presenting cells, type 3 innate lymphoid cells (ILC3), and B cells [60,61]. SCFA also supports defense responses in systemic tissues such as the spleen and lymph nodes. Finally, SCFAs also have the ability to modulate cytokines in immune cells. Butyrate can increase the production of IL-22 by ILC3 and CD4+ T cells. It also plays a role in increasing the cytotoxicity of CD8+ T cells [49]. In particular, among SCFAs, butyrate has systemic anti-inflammatory properties by altering adhesion, migration, and cytokine expression of immune cells, thus influencing cellular processes such as proliferation, activation, and apoptosis [62]. In addition, it is often associated with immune regulation mainly through induction of T-reg cells [63].

Several hypotheses have been made about the mechanism of action of short-chain fatty acids. One of them is that SCFAs can affect histone deacetylase (HDAC). SCFAs are thought to contribute to the regulation of HDAC, affecting cell adhesion, immune cell migration, cytokine production, chemotaxis, and programmed cell death [64]. These properties of SCFA can enhance the immunomodulatory effect [65]. As a result, they could modulate the development of tumors. The effect of SCFA on gastrointestinal and lung cancer has been studied. Yang et al. [66] demonstrated the hypothesis that intestinal bacterial imbalance in diabetic patients induces tumorigenesis through low microbial SCFA levels, enhanced microbial migration, and increased inflammatory response.

Studies on SCFA have been reported most frequently in colorectal disease. One of the characteristics of inflammatory bowel disease (IBD) is that fecal SCFA concentration is significantly lower than that in the control group [67]. Enema with mixed SCFAs (acetate, propionate, butyrate) has been shown to be effective in improving symptoms of patients with ulcerative colitis [68]. Relationships of SCFAs with other diseases have also been reported. A negative correlation of SCFAs with the risk of obesity, insulin resistance, and type 2 diabetes has been reported [69] because SCFAs can affect fatty acid, glucose, and cholesterol metabolism [70].

In urology, the association between SCFA and disease has been the most studied in urolithiasis. Calcium oxalate (CaOx) stones are the most common type of urolithiasis. They are thought to be associated with SCFA and inflammation. Liu et al. [71] have conducted a case-controlled study using stool samples from kidney stone patients and healthy controls. *Blautia*, *Anaerostipes*, *Coprococcus*, *Fusobacterium*, *Ruminococcus*, and *Lachnospiraceae* were found to be fewer in recurrent stone patients than in the control group. Their metabolites were mainly SCFAs [72]. In an experiment using mice, administration of SCFA reduced the formation of CaOx crystals in kidneys and urinary oxalate levels, but increased SCFA-producing bacteria and SCFA in the colon [73].

However, oral supplementation of high doses of propionate in rats impaired the insulin signaling system suggesting that overproduction of acetate using intestinal microbes could lead to fatty liver [74]. One study [75] has also suggested that overproduction of acetate using intestinal microbes can lead to fatty liver. This suggests that oral administration of SCFAs might not be beneficial, whereas a balanced production of SCFA by the intestinal microflora is important.

Recently, Worby et al. [21] have performed a study on recurrent UTI and found that microbial abundance and SCFA producers, especially butyrate-producing bacteria, are significantly depleted in the gut microbiome of patients with recurrent infection compared to those in controls. Although the intestinal and bladder dynamics of *Escherichia coli* were similar between cohorts, the rUTI strain that colonized the intestine was not cleared for a long time by antibiotic treatment. They concluded that a distinct immune response to bacterial invasion of the bladder is potentially mediated by the gut microbiome [76]. This is evidence for the gut–bladder axis. It is presumed that SCFA plays an important role. SCFAs can also directly act on the brain and play an important role in the axis described above [77]. Another study has shown that the normal microbiome can protect the host-associated niche from pathogens by controlling abiotic factors and that an environment unfavorable for maintaining a normal microbiome or dysbiosis can make it easier for microbiota to pass through the perianal–urogenital pathway [78]. Dysbiosis can make individuals become vulnerable to UTI and is also thought to play a role in the pathogenesis of UTI.

Studies about dysbiosis of gut microbiota and their effects on prostate disease have been limited. Studies on the effect of SCFA on the prostate are also lacking. The effect of the gut microbiota on the prostate was mainly related to chronic inflammation [79]. Chronic prostate inflammation predisposes to BPH and prostate cancer and may be associated with low-grade systemic inflammation [79]. Men with inflamed areas of the benign prostate biopsy tissue were 1.78 times more likely to be positive for prostate cancer than those without inflammation [80]. Inflammation of prostate shares many of the genomic and protein alterations seen in high-grade prostate cancer, indicating an association between inflammation and cancer [81]. Disturbed gut microbiota appears to contribute to the pathogenesis of chronic systemic inflammation without directly affecting the prostate [82]. As mentioned earlier, SCFAs also play an important role in the immune system. Recently, there was a report analyzing SCFA in BPH patients. Ratajczak et al. [70] evaluated the SCFAs composition in stool samples of study participants using gas chromatography. They reported that SCFA levels were associated with BPH, suggesting that SCFA may be important in future BPH studies.

Although it is true that SCFAs are important in demonstrating a link between the gut microbiome and the host, the underlying mechanism remains unclear. A better understanding of these interconnections will aid in disease pathogenesis and treatment.

## 4. Strength and Limitation of This Study

Recently, as the evidence for the importance of the gut microbiome increases, crosstalk with other organs, such as the gut–brain axis, is being studied. However, crosstalk studies between the urinary microbiome and other organs are increasing, but very sporadically. Accordingly, we organized and summarized the relationship between the urinary microbiome and other organs reported so far as an axis system. Additionally, the mediator of SCFA and the inflammasome was presented as a link between the urinary microbiome and other organs. The strength of this study is that it was able to more precisely suggest the relationship between the vague microbiome and disease.

However, as mentioned above, there are few studies on this yet, and clinical data are limited. It is expected that many studies will support this in the future.

## 5. Conclusions

The recently reported bladder–gut–brain axis, gut–bladder axis, gut–vagina–bladder axis, and gut–kidney axis have provided important clues to understand the pathophysiology of disease that was not previously known. There is also increasing evidence that SCFAs play an important role in axis crosstalk including the relationship between UTI and the intestinal microbiome. In the future, more studies will be conducted on the role of axis substances (SCFAs and inflammasome, etc.) in various urinary tract diseases, and it will help to develop novel treatment methods.

## Figures and Tables

**Table 1:** Short-chain fatty acids and their bacterial producers.

Main Types of SCFAs	Chemical Structure of Anions and Molecular Weight	Bacterial Producers	Ref.
Acetate	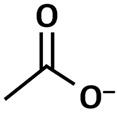 60.05	*Akkermansia muciniphila, Bacteroides* spp., *Bifidobacterium* spp., *Prevotella* spp., *Ruminococcus* spp., *Blautia hydrogenotrophica*, *Clostridium* spp., *Streptococcus* spp., *Coprococcus* spp.,	[51,52]
Butyrate	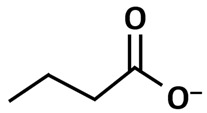 88.11	*Anaerostipes* spp., *Coprococcus catus, Eubacterium rectal, Eubacterium hallii, Faecalibacterium prausnitzii, Roseburia* spp., *Coprococcus comes, Coprococcus eutactus, Anaerostipes* spp., *Coprococcus catus*, *Lactobacillus salivarius* spp. *Clostridium butyricum, Ruminococcaceae*	[51,53,54]
Propionate	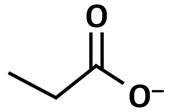 74.00	*Bacteroides* spp., *Dialister* spp., *Veillonella, Roseburia, Megasphaera elsdenii, Coprococcus catus, Salmonella* spp., *Roseburia inulinivorans*, *Ruminococcus obeum, Phascolarctobacterium succinatutens inulinivorans*,	[51,53]
